# Multiscale Structural Evolution and Its Relationship to Dielectric Properties of Micro-/Nano-Layer Coextruded PVDF-HFP/PC Films

**DOI:** 10.3390/polym12112596

**Published:** 2020-11-05

**Authors:** Jie Wang, Daniel Adami, Bo Lu, Chuntai Liu, Abderrahim Maazouz, Khalid Lamnawar

**Affiliations:** 1 Key Laboratory of Materials Processing and Mold (Ministry of Education), National Engineering Research Center for Advanced Polymer Processing Technology, Zhengzhou University, Zhengzhou 450002, China; jjwangnet@163.com (J.W.); ctliu@zzu.edu.cn (C.L.); 2 CNRS, UMR 5223, Ingénierie des Matériaux Polymères, INSA Lyon, Université de Lyon, F-69621 Villeurbanne, France; daniadami_@hotmail.com (D.A.); abderrahim.maazouz@insa-lyon.fr (A.M.); khalid.lamnawar@insa-lyon.fr (K.L.); 3 Hassan II Academy of Science and Technology, 10100 Rabat, Morocco

**Keywords:** micro-/nano-layer coextrusion, multilayer films, multiscale structure, dielectric properties

## Abstract

An understanding of the structural evolution in micro-/nano-layer co-extrusion process is essential to fabricate high-performance multilayered products. Therefore, in this work, we reveal systematically the multiscale structural development, involving both the layer architecture and microstructure within layers of micro-/nano-layer coextruded polymer films, as well as its relationship to dielectric properties, based on poly(vinylidene fluoride-*co*-hexafluoropropylene) (PVDF-HFP)/polycarbonate (PC) system. Interestingly, layer architecture and morphology show strong dependences on the nominal layer thicknesses. Particularly, with layer thickness reduced to nanometer scale, interfacial instabilities triggered by viscoelastic differences between components emerge with the creation of micro-droplets and micro-sheets. Films show an enhanced crystallization with the formation of two-dimensional (2D) spherulites in microlayer coextruded systems and the oriented in-plane lamellae in nanolayer coextruded counterparts, where layer breakup in the thinner layers further changes the crystallization behaviors. These macro- and microscopic structures, developed from the co-extrusion process, substantially influence the dielectric properties of coextruded films. Mechanism responsible for dielectric performance is further proposed by considering these effects of multiscale structure on the dipole switching and charge hopping in the multilayered structures. This work clearly demonstrates how the multiscale structural evolution during the micro-/nano-layer coextrusion process can control the dielectric properties of multilayered products.

## 1. Introduction

Multilayer polymer films have been increasingly used in electrics, energy, display devices, packaging, construction and other applications. Methods of fabricating polymer multilayer films generally include layer-by-layer assembly (LbL) [1], and multilayer coextrusion technology [2,3]. Among them, the multilayer coextrusion technology has aroused widespread interest over the past 20 years, because it has become a reliable technology for continuous production of micro- and nanolayers. Different from layer-by-layer assembly, multilayer coextrusion is a top-down method that can manufacture thousands of layers of films with layer thicknesses controllable down to nanometer scale, thus endowing the final films with high degree of flexibility, tunable gas/liquid barrier [4], mechanical [5,6], optical [7], and electrical properties [8,9].

Generally, a stable and continuous multilayer morphology in films is necessary to achieve the tailor-made end-use properties that are superior to those of conventional polymer blend films. Many efforts have been, thus, made to prepare microlayer and nanolayer films with a great number of layers alternating of polymer pairs, such as polystyrene (PS)/poly(methyl methacrylate) (PMMA) [10], polycarbonate (PC)/PMMA [11], PC/polyvinylidene fluoride (PVDF) [12], etc. Although, many polymer pairs could be combined to produce multilayer films via layer multiplying coextrusion technology, layer instabilities can occur under the laminar flow conditions, which deteriorates the layer continuity and uniformity [13,14]. This concern holds particularly true for polymer pairs with a large difference in rheological properties between components [2]. Also, it has been observed that below a certain layer thickness normally of several nanometers, layers tend to lose their integrity with severe interfacial distortions [15]. Some polymer layers can even break into nanosheets and nanodroplets. The presence layer instabilities and layer breakup can greatly influence the properties of films [15]. Apart from the macroscopic layer structure, the microstructure inside the layers are also important in defining the macroscopic properties of multilayer products. With a number of layers multiplied in a limited space, various microstructure and dynamics from molecular aggregation within layers, including the macromolecular alignment, crystallization, and glass transition, can be developed with the decrease in layer thicknesses [16,17,18]. For example, a confined crystallization with highly oriented crystals stacked between layers was recently reported by reducing the layer thicknesses, which resulted an enhanced dielectric and other physicochemical properties [19,20,21]. Despite of the huge importance of multilayer films in the daily life, the evolutions in both the macroscopic layer structure and microstructure inside layers are far from being completely understood, as well as their effects on the resulting macroscopic properties. The question of understanding how the multiscale structure develops all along the multilayer coextrusion processing line then emerges as a crucial issue, in order to optimize the process parameters and manufacture multilayer films with excellent properties.

Therefore, the objective of this present work is to demonstrate the development of multiscale structure along the multilayer coextrusion process and its influence on the resulting dielectric properties. To this end, films alternating components of poly(vinylidene fluoride-*co*-hexafluoropropylene) (PVDF-HFP) and polycarbonate (PC) were fabricated by micro-/nano-layer coextrusion process. The evolution in the layer structure and microstructure of layers with varying the number of layers or layer thicknesses was investigated. By combining a high dielectric constant polymer of PVDF-HFP with super electrochemical properties [22,23,24], and a linear dielectric polymer of PC with low dielectric loss and high breakdown strength, dielectric properties of the obtained films were further investigated. The relationship between the multiscale structure and the final dielectric properties was thus established, and the corresponding mechanisms was elucidated. This work will give some new guidelines for controlling the multiscale structure development and macroscopic properties of multilayer products from multilayered assembly processing.

## 2. Experimental Section

### 2.1. Materials

Two kinds of polymers were used in this work. Poly(vinylidene fluoride-*co*-hexafluoropropylene) (PVDF-HFP, Kynar Flex 2500-20, ARKEMA, Colombes, France) with weight-average molecular weight *M*_w_ of 500,000 g/mol and melt flow index of 5.8 cm^3^/10 min at 230 °C/3.8 kg was supplied by Arkema (Colombes, France). PC (Calibre 303EP) with *M*_w_ of 23,000 g/mol and melt flow index of 5.8 cm^3^/10 min at 300 °C/1.2 kg, were kindly provided by Trinseo (Stade, Germany). Both virgin polymers were in the granular pellet form. The glass transition temperatures of PVDF-HFP and PC are −34, and 150 °C, respectively, as determined using a differential scanning calorimetry. All the pristine products were dried in a vacuum oven at 80 °C for 48 h before usage.

### 2.2. Sample Preparation

**Figure 1:**
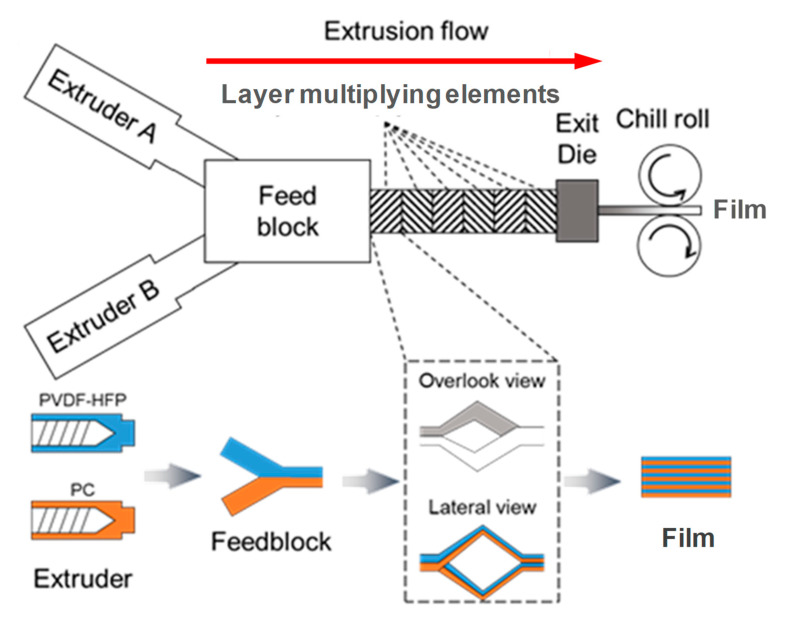
Schematic of the layer multiplying process in the multilayer coextrusion system.

PVDF-HFP/PC multilayer films were prepared using a homemademultilayer coextrusion system that is capable of preparing microlayer and nanolayer films. As schematically illustrated by Figure 1**,** this setup is composed of two single-screw extruders (A and B), a feedblock, a set of layer multiplying elements, an exit die and a thermally regulated chill roll. The layer multiplying element used here has a constant cross-sectional area. More details about this multilayer coextrusion system has been given in our recent report [25]. During processing, two component polymer melts of PVDF-HFP and PC are extruded from two extruders, and brought together at the feedblock, followed by going through a series of layer multiplying elements. Each element multiplies the melt while keeping the total melt thickness constant, thus doubling the number of layers and reducing the individual layer thickness by a factor of 2. During coextrusion process, the extruders, multipliers and die temperatures were prescribed to 250 °C. Films containing the nominal number layers of 2-16384 layers were fabricated by a given number of multipliers (Table 1). The as-prepared multilayer films were abbreviated as “*NL*” where N represents the nominal number of layers. Films were collected by a chill roll at a temperature of 80 °C with a negligible speed to take them without stretching. The nominal layer thicknesses were calculated by the total thickness divided by the nominal number of layers and listed in Table 1. Layer thicknesses of all films were further determined by the morphological investigations.

### 2.3. Characterizations

#### 2.3.1. Rheological Measurements

Linear viscoelasticity of PVDF-HFP and PC was evaluated using a shear rheometer (DHR-2, TA Instruments, New Castle, DE, USA) with a 25 mm parallel-plate geometry at different temperatures from 220 to 260 °C under a nitrogen atmosphere. Dynamic frequency sweep tests were performed from the angular frequency of 628–0.05 rad/s and under a strain of 5%.

#### 2.3.2. Morphological Observations

Cross-sectional morphology of as-coextruded films were observed by an atomic force microscope (AFM, Bruker Multimode 8, Santa Barbara, CA, USA) at room temperature. Before our observation, the films were embedded in the standard epoxy, and cured at room temperature for overnight. A flat and smooth cross section of the cured film was obtained by cryo-ultramicrotoming at −80 °C with a diamond knife blade. Phase and height images of the cross sections were taken during observations.

#### 2.3.3. Differential Scanning Calorimetry (DSC)

Thermal properties of the as-coextruded films were examined using a differential scanning calorimeter (DSC, Q20, TA Instruments, New Castle, DE, USA) under a nitrogen atmosphere. Specimens around ~5 mg taken from films were loaded into the DSC aluminum pans. Samples were first heated from −80 to 240 °C and then equilibrated at 240 °C for 3 min, followed by being cooled to −80 °C. Both the heating and cooling rates were set as 10 °C/min. The crystallinity (*X_c_*) of PVDF-HFP in films was determined from the enthalpy of fusion (Δ*H_m_*) in the heating scan according to,

(1) $$X_{c}=\frac{\Delta H_{m}}{w\Delta H_{m}^{0}}\times 100\%$$

where *w* is the weight fraction of PVDF-HFP, and $\Delta H_{m}^{0}$ is the theoretical melting enthalpy of 100% crystalline polymer of PVDF-HFP, estimated as 104.6 J/g from the literature data [26].

#### 2.3.4. X-ray Analyses

The crystalline morphology and structure in as-coextruded films were explored using two-dimensional wide-angle X-ray diffraction (WAXD, D8 Discover, Bruker, Karlsruhe, Germany) and small-angle X-ray scattering (SAXS, NanoSTAR-U, Bruker, Karlsruhe, Germany). The X-ray wavelength was 0.154 nm with Cu Kα radiation source. Signals were obtained by aligning the incident X-ray beam parallel to the extrusion direction (ED) of the film.

#### 2.3.5. Fourier Transform Infrared Spectroscopy (FTIR)

Fourier transform infrared spectroscopy was carried out using an infrared spectrometer (Nicolet 6700, Thermo Fisher Scientific, Waltham, MA, USA). Infrared spectra were collected in transmission mode at a resolution of 4 cm^−1^ with 64 scans. A thin slice about 4 μm in thickness was cut from the as-extruded films using a Rotary microtome (Leica RM2235, Leica Microsystems GmbH, Wetzlar, Germany) at room temperature. Samples, same to those for morphological measurements, were embedded in the standard epoxy and cured at room temperature prior to cutting.

#### 2.3.6. Dielectric Measurements

Dielectric properties of as-coextruded films were measured using a precision LCR meter (Agilent E4980A, Agilent Technologies Inc., Penang, Malaysia) equipped with an Environmental Test Chamber (ETC, TA Instruments, New Castle, DE, USA) for temperature control. The silver electrode is applied to both sides of the film samples with a diameter of 25 mm. Frequency scans from 20 Hz to 2 MHz were carried out with a constant voltage of 1 V. For temperature scans, temperature was ramped from −60 to 150 °C at a rate of 5 °C/min. All tests were repeated at least three times with fresh samples to ensure the reproducibility.

## 3. Results and Discussion

### 3.1. Viscoelastic Ratios between PVDF-HFP and PC

**Figure 2:**
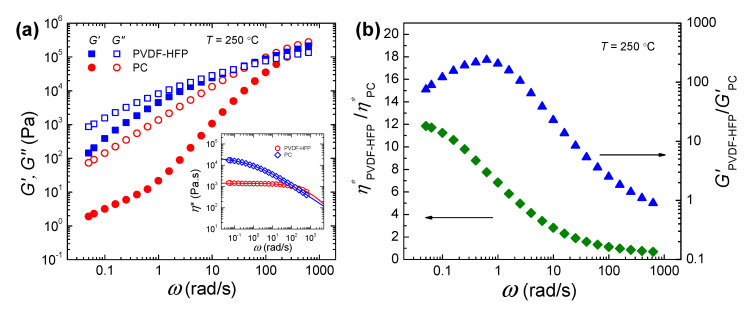
(**a**) Comparison of storage modulus (*G′*) and loss modulus (*G″*) against angular frequency for PVDF-HFP and PC at 250 °C. The inset in (**a**) shows the complex viscosity versus frequency. (**b**) Viscosity and elasticity ratios of PVDF-HFP to PC against angular frequency at 250 °C.

It is known that the melt viscosity and/or elasticity ratios among components are highly important in determining the layer architecture and uniformity for multilayered polymer structures. To determine the viscoelastic ratios, the linear viscoelasticity of PVDF-HFP and PC were investigated using dynamic shear rheology at the processing temperature of 250 °C (Figure 2a), and the frequency dependent viscosity and elasticity ratios between two polymers were computed accordingly (Figure 2b). PVDF-HFP shows the higher elasticity with the larger storage modulus relative to PC within the measured frequency range. Also, PVDF-HFP is more viscous with the larger complex viscosity and loss modulus than PC, and it shows the more pronounced shear thinning (see inset of Figure 2a). Due to the different frequency dependencies of PVDF-HFP and PC a decrease in viscosity and elasticity ratios can be noticed by increasing the frequency. Typically, the shear rate range of the coextrusion process lies between 1 and 10 s^−1^ [27]. Therefore, assuming the validity of Cox-Merz rule [28], the viscosity ratio of PVDF-HFP to PC within 1−10 s^−1^ is estimated to be ranging from 2.8 to 6.8, while the elasticity ratio is from 15 to 200 depending on the shear rates of coextrusion processing window (Figure 2b).

### 3.2. Layer Morphology of As-Coextruded Films

**Figure 3:**
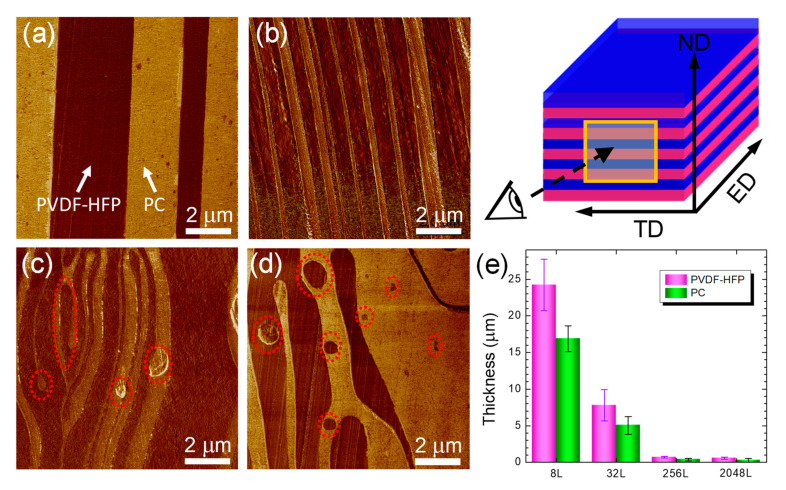
AFM phase images for PVDF-HFP/PC films: (**a**) 32 L, (**b**) 256 L, (**c**) 2048 L and (**d**) 16384 L. (**e**) is the statistical layer thicknesses determined by AFM analysis. Regions inside the dashed circles in (c) and (d) indicate the broken layers. The scheme appended in the upper right corner shows the configuration of observed cross-sections relative to the extrusion direction (ED), transverse direction (TD), and normal direction (ND) of the film.

The layer structure and uniformity in as-coextruded PVDF-HFP/PC films were investigated using AFM. Figure 3a–d display the morphological features of cross-sections of films with various nominal number of layers. In the phase images, the dark phase represents PVDF-HFP and the bright phase represents PC, due to the higher modulus of PC than PVDF-HFP at room temperature. As visualized by AFM, continuous PVDF-HFP and PC layers are noticeable for films with nominal number of layers ranging from 2 to 256. Using image analysis, the average layer thickness for both components were determined (Figure 3e). In this instance, for the 32 L film PVDF-HFP and PC layer thickness were identified to be 7.79 ± 2.12 μm, and 5.05 ± 1.22 μm, respectively. The thicker PVDF-HFP layers than PC layers are ascribed to the higher viscosity and modulus during layer multiplication process. Constituent layer thicknesses were further decreased by increasing the number of layers, e.g., 703 ± 121 nm for PVDF-HFP and 365 ± 192 nm for PC in the 256 L film. However, at the nominal layer thicknesses below 160 nm, about half of the layers remains continuous and some layers even broke up into micro-sheets. It is evident that these elongated micro-sheets are still parallel to the survived continuous layers, even when some of the micro-sheets coalesced into droplets (see dashed circles in Figure 3c–e). Those micro-sheets and droplets result from interfacial instabilities in the unstable continuous layers that are triggered by the melt viscosity and elasticity differences between two constituent polymers during layer multiplying coextrusion process [2,15]. With the further decrease in the layer thicknesses, the layers are prone to having a gradual breakup process. At 25 nm nominal layer thickness (i.e., 16384 L film, Figure 3d), almost no continuous layers can be discerned across the film. Such a nominal layer thickness is close to the critical layer thickness (~10 nm) for the occurrence of layer breakup as recently reported for immiscible PS/PMMA multilayer systems [29]. It is also worthwhile that at the locations where layer breakup occurred (Figure 3d), some droplets display a diameter up to ~1.0 μm, and some sheets have a thickness of several microns, of which the sizes are unprecedently larger than the expected nominal layer thickness. Similar morphological feature has been also observed in PMMA/PS nanolayer films when subjected to a post-annealing to the molten state [10]. The larger sizes of droplets and sheets could be explained by the physical properties of polymers. PVDF-HFP is a semicrystalline polymer with a melting temperature of 124 °C and crystallization temperature of 92 °C, while PC is an amorphous polymer with a glass transition of 150 °C. It could be expected that phase coalescence of thin layers took place as the polymer flow exited the extrusion die (250 °C), thus leading to the larger sized micro-droplets and micro-sheets.

### 3.3. Microstructure Evolutions in As-Coextruded Films

**Figure 4:**
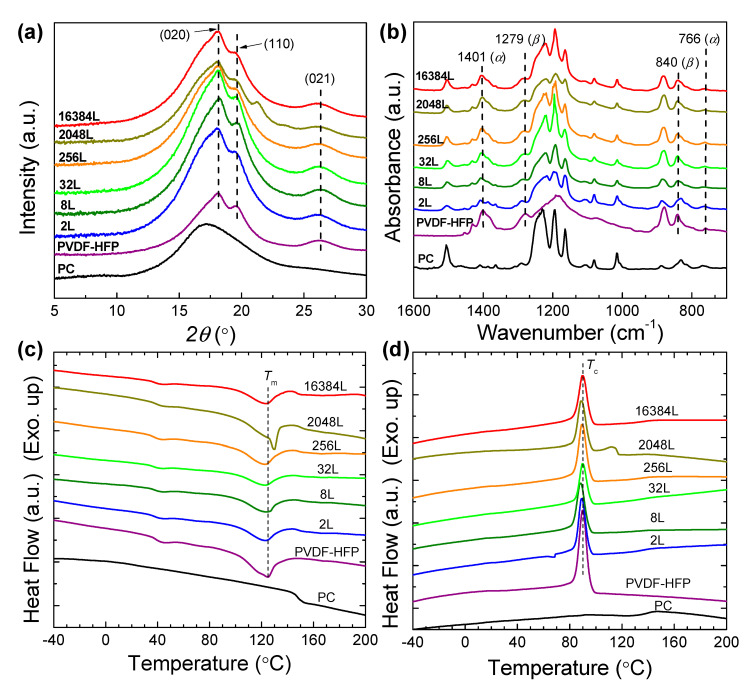
(**a**) WAXD profiles for coextruded PVDF-HFP/PC films varying the nominal number of layers. The intensity was normalized with film thicknesses. (**b**) FTIR spectra of PVDF-HFP/PC films. DSC thermographs of; (**c**) heating scan; and (**d**) cooling scan for coextruded PVDF-HFP/PC films.

**Figure 5:**
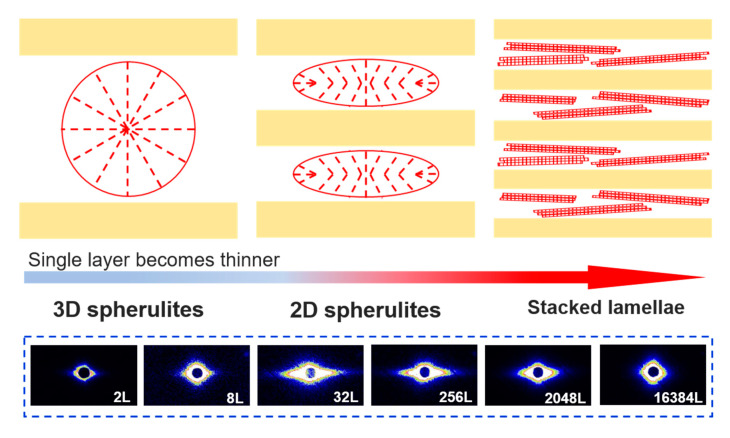
Schematic of the morphology evolution of PVDF-HFP crystallization with the decrease in layer thicknesses from micro- to nanoscale. The panel appended at the bottom shows the SAXS patterns recorded with X-ray beam parallel to the extrusion direction for coextruded PVDF-HFP/PC films.

The microstructure inside the as-coextruded PVDF-HFP/PC films was further investigated. Figure 4a shows the X-ray diffraction profiles captured by WAXD for all films. In all as-extruded films, PVDF-HFP crystallized typically in the dominated *α*-phase as noticed from the diffraction peaks indexed as (020), (110) and (021) planes [30]. Further analysis of crystalline polymorphism using FTIR reveals that strong *α*-phase with some traces of *β*-phase coexists in these films as evidenced by peaks at 766 and 1041 cm^−1^ for the *α*-phase, and 1279 and 840 cm^−1^ for the *β*-phase (Figure 4b) [31]. Such polar *β*-phase crystals that are electrically active phases are beneficial for application in dielectric and piezoelectric devices. The formation of *β*-phase should be related to the strong polarized interatomic C-F bonds from the extreme electronegativity of the fluorine atoms compared to that of carbon atoms in the copolymer of PVDF-HFP [32]. It is also interesting that an increase in the diffraction intensity is noticeable with increasing the nominal number of layers, indicating an enhanced crystallization of PVDF-HFP at the higher number of layers. This observation was further validated by the crystallinity (*X*_c_) determined by DSC analysis (Figure 4c, Table 2). Quantitatively, the crystallinity of coextruded films is gradually increased to be 20.4% in the 2048 L film and then further reduced to be 13.3%, which is clearly larger than that of pure PVDF-HFP. The observed increase in crystallinity is explained by the microstructure evolution of crystallization in the layered systems. As illustrated by the scheme in Figure 5, for microlayer films the crystals have relatively larger space to crystallize into three-dimensional (3D) spherulites. However, with the decrease in layer thicknesses, these spaces allowing for crystallization are reduced, which would result in the formation of 2D disc-like oriented spherulites (see the bottom panel of Figure 5). As the layer thickness is further reduced to nanoscale at the higher number of layers, the highly oriented in-plane lamellae will be formed between two neighboring rigid PC layers [16,18]. These stacked lamellae are believed to have the more crystallinity than spherulites composed of crystallization and interlamellar amorphous regions. This could explain the increased crystallinity at the higher number of layers.

It is also surprising to view that the melting behavior for the 16,384 L film resembles those for 2 L to 256 L films (Figure 4c). For nanolayer coextruded films with a larger amount of layer breakup, the broken thin layers and sheets would relax and coalesce into a larger phase structure like droplets before melt crystallization as discussed earlier. The confinement of rigid PC layers on the crystallization of PVDF-HFP would be therefore alleviated, which is confirmed by the reduced structural orientations shown by the SAXS pattern of 16384 L film (see the bottom panel of Figure 5). This led to the formation of crystallization structure close to that in microlayer films, as evidenced by the similar crystallinity as the latter cases (Table 2). This argument was further supported by checking the crystallization behaviors of films during the DSC cooling scan after eliminating all layer structures through an annealing at an elevated temperature of 240 °C for 3.0 min to (Figure 4d). Notably, the crystallization temperatures (*T*_c_) for all annealed films almost coincides with these of microlayers and bulk PVDF-HFP component (Table 2). This, in turn, suggests that different crystallization behaviors of PVDF-HFP in a layered system from that in a blend-like system.

### 3.4. Dielectric Properties of As-Coextruded Films

**Figure 6:**
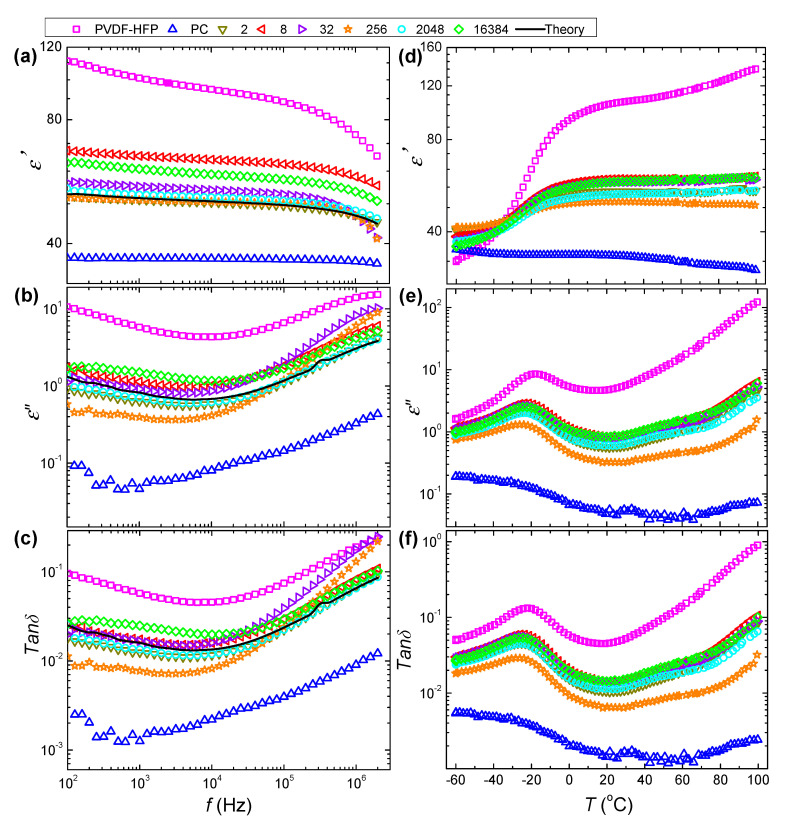
(**a**) Storage permittivity, (**b**) loss permittivity, and (**c**) loss tangent spectra versus frequency at 30 °C for PVDF-HFP/PC films. (**d**–**f**) Temperature dependent dielectric spectra measured at 1 kHz. Solid lines in (**a**–**c**) are theoretical predictions for layered dielectrics without interphase layers.

Coextruded multilayer films are known as the promising materials in dielectric capacitor applications. Therefore, dielectric properties of coextruded PVDF-HFP/PC films were evaluated using dielectric spectroscopy. Figure 6a-c compares the dielectric spectra as a function of frequency for as-coextruded PVDF-HFP/PC films measured at the room temperature (30 °C). The corresponding dielectric spectra versus temperature at the tested frequency of 1 kHz are plotted in Figure 6d–f. Clearly, the dielectric properties of these films show a strong dependency on the nominal number of layers. As shown in Figure 6a, the values of *ε′* (dielectric constant *k* = *ε′/ε*_0_ with *ε*_0_ being the vacuum permittivity) at 30 °C for PVDF-HFP/PC films fall in between neat polymers. The *ε′* is gradually improved by increasing the temperature due to the fact that dipoles become more activated upon heating. To clearly demonstrate the dependency of dielectric properties on the nominal number of layers, the theoretical spectra of the multilayer system are also calculated (dashed lines in Figure 5) according to the formula as detailed in our recent study [25,33]. It is obvious that the value of *ε′* is continuously increased with nominal number of layers increasing up to 8. Herein, the increased *ε′* is related to the dipole polarizations contributed by the effects from crystallizations and molecular orientations. As aforementioned, increasing the nominal number of layers enhanced the crystallizations that would lead to less mobile dipoles, which are detrimental for improving dipole polarization. Instead, the improved structural orientations with the reduction in the layer thicknesses result in the alignment of the *c*-axis of crystals along the film in-plane direction, which facilitates the preferential switching of dipole moments parallel to the electric field. Therefore, such improvement in *ε′* is ascribed primarily to the enhanced molecular orientations. However, we can also note that *ε′* is further suppressed with increasing the nominal number of layers to 16,384. This should be caused by the reduced orientations by the presence of layer breakup, as demonstrated above. It is also worth mentioning that the dielectric performance obtained in this study is higher than that reported in the literature. Taking the 32 L film as an example, the dielectric constant k and dielectric loss tanδ are 6.7 and 0.01, at 1000 Hz, respectively, which is superior to literature values for 32 L film with K being 4.5 and tanδ being 0.02 [34,35]. This should be attributed to the higher structural orientation in the multilayer system of this study.

**Figure 7:**
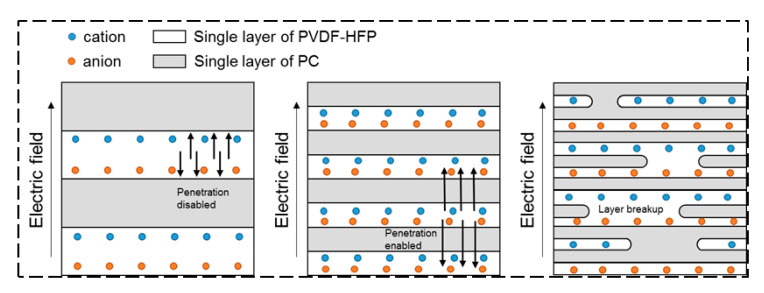
Schematic illustration of the interfacial polarization of space charges in coextruded PVDF-HFP/PC films varying layer thickness and structure under an external electric field.

It is also worth mentioning that the dielectric loss of the microlayer films (i.e., 2 L to 256 L) are lower than that of 2048 L and 16,384 L films. This could be interpreted by invoking the structural evolutions in PVDF-HFP/PC films. As known, dielectric loss for polymer dielectrics are mainly related to the hopping of charge carriers and the energy dissipation from the switching of dipoles [36]. As schematically shown in Figure 7, under the action of an external electric field, charge ions, free electrons and polarized impurity ions in the more conducting component of PVDF-HFP will migrate and accumulate at the PVDF-HFP/PC interfaces. Namely, the more insulating PC layers act as charge-blocking layers to form interfacial polarization. The interfacial charges thus alter the local electric field, hinder the hopping of electrons, and eventually minimize the leakage current going through the whole films. Therefore, the thicker the PC layer, the more interface charges will be effectively blocked. However, when a large number of layers are broken in nanolayer extruded films, the charger carriers can move easily across physical thickness of film, resulting in a large increase in dielectric loss (e.g., 16,384 L). Also, for nanolayer coextruded films, with the occurrence of layer breakup the energy dissipation from the switching of dipoles is higher due to the decreased molecular orientations along the in-plane direction as aforementioned. These effects thus accounts for the higher dielectric loss in 2048 and 16,384 L films. Therefore, it is concluded that the dielectric performance of multilayer films is strongly dependent on the layer architecture and microstructure. A better control of multilayer structure via processing offers a strategy for tailoring their dielectric properties.

## 4. Conclusions

In summary, we investigated the multiscale structural development and its relationship with the dielectric properties of micro-/nano-layer coextruded polymer films based on PVDF-HFP/PC system. Multiscale structural evolutions involving both the layer architecture and microstructure within layers were revealed systematically. The layers were stable and continuous for microlayer coextruded films with nominal layer thickness. Whereas, for nanolayer coextruded films at nominal layer thickness below 160 nm, layer integrity was reduced by interfacial instabilities triggered by viscoelastic differences between component melts. Layers even broke into micro-droplet and micro-sheets due to the coalescence of thin layers. Besides, with the reduction in nominal layer thickness, films displayed an enhanced crystallization, with the formation of 2D oriented spherulite structure in microlayer coextruded systems and highly oriented in-plane lamellae in nanolayer coextruded counterparts. Layer breakup in thinner layers further resulted in the crystallization and structural orientation similar to that in microlayer films, which was attributed to the relaxation and phase coalescence of thin layers during processing. Furthermore, dielectric properties of films were strongly dependent on these multiscale structures. The gradually increased storage permittivity with reducing layer thickness was ascribed to the enhanced molecular orientations that could facilitate the dipole switching. The lower storage permittivity in thin layers with breakup was caused by the reduced orientations. In addition, the dielectric loss of microlayer coextruded films was lower than that of nanolayer coextruded analogues. This was due to the increased hopping of charge carriers and the higher energy dissipation from dipole switching when layers were broken. The results of this study will enable a better understanding of the multiscale structure evolution in micro-/nanolayer coextrusion to optimize the target macroscopic properties.

## Figures and Tables

**Table 1:** Characteristics of coextruded PVDF-HFP/PC films.

Samples	No. of Multipliers (n)	No. of Layers (N)	Total Film Thickness (μm)	Nominal Layer Thickness (nm)
2 L	0	2	243	121,250
8 L	2	8	245	30,630
32 L	4	32	248	7730
256 L	7	256	313	1220
2048 L	10	2048	325	158
16,384 L	13	16,384	415	25

**Table 2:** Melting temperature (*T_m_*), enthalpy of fusion (Δ*H_m_*), crystallinity (*X_c_*) and crystallization temperature (*T_c_*) obtained from the first heating scan of DSC.

Samples	*T_m_* (°C)	Δ*H_m_* (J/g)	*X_c_* (%)	*T*_c_ (°C)
Pure PVDF-HFP	124.81	13.57	12.9	92.08
Pure PC	-	-	-	-
2 L	122.54	8.77	13.9	91.94
8 L	123.14	9.098	14.4	91.52
32 L	122.31	10.69	16.9	91.78
256 L	122.24	10.78	17.1	91.26
2048 L	128.69	12.85	20.4	91.13
16,384 L	123.02	8.35	13.3	91.72
